# Current status of and barriers to the treatment of advanced-stage liver cancer in China: a questionnaire-based study from the perspective of doctors

**DOI:** 10.1186/s12876-022-02425-4

**Published:** 2022-07-24

**Authors:** Qiaoxin Wei, Haiyang Zhou, Xinhui Hou, Xiaoping Liu, Sisi Chen, Xueying Huang, Yu Chen, Mei Liu, Zhongping Duan

**Affiliations:** 1 Department of Oncology, Beijing You’an Hospital, Capital Medical University, Beijing, 100069 China; 2 Beijing iGandan Foundation, 103-5C16-1, 1F, Building 1, No. 16, South Third Ring West Road, Fengtai District, Beijing, 100069 China; 3 Fourth Department of Hepatology Center, Beijing You’an Hospital, Capital Medical University, Beijing, 100069 China

**Keywords:** Liver cancer, Doctor satisfaction, Barriers, Questionnaires, Cross-sectional study

## Abstract

**Background:**

Liver cancer is a severe public health problem worldwide, and it creates a relatively higher disease burden in China than in the Western world. Despite achieving notable progress in China, potential differences in some aspects of medical services for liver cancer may persist across different regions and hospitals. This warrants serious consideration of the actual status of and barriers to liver cancer treatment. We intended to explore the present status of and obstacles in liver cancer treatment especially for advanced-stage liver cancer.

**Methods:**

In February 2021, a national multicenter cross-sectional study was conducted among 1500 doctors from 31 provinces of mainland China using a self-administered online questionnaire. Participants completed the questionnaire about their general information, perspectives on the current status of liver cancer treatment, and expectations for future treatment. Chi-square and logistic regression analyses were performed to explore the differences associated with the regions, doctors’ professional ranks, and hospital levels.

**Results:**

Treatment conditions, medications, and treatment strategies were inconsistent across different economic regions and hospital of different levels. With respect to obstacles in treatment, 76.6% of the doctors were unsatisfied with the current treatment for liver cancer. Important factors that influenced their satisfaction with the treatment for liver cancer included early diagnosis and the disclosure of true conditions to patients.

**Conclusions:**

There persists differences in the treatment of liver cancer in China, besides barriers to treatment. More attention should be paid to the detection and treatment of liver cancer and the propagation of novel progress among doctors in underdeveloped areas.

**Supplementary Information:**

The online version contains supplementary material available at 10.1186/s12876-022-02425-4.

## Introduction

As a major public health issue, liver cancer is the sixth most diagnosed cancer and the third leading cause of cancer-related deaths worldwide [1]. The incidence of liver cancer in China is higher than that in other countries principally due to high prevalence of hepatitis B [1, 2]. Approximately 4,292,000 people in China are newly diagnosed with liver cancer each year, and approximately 2,814,000 patients die from the disease annually [3]. The condition is often detected at an advanced stage, making the treatment extremely difficult, and the therapeutic outcomes are far from expectations [4]. Therefore, liver cancer is a serious threat to the lives and health of the Chinese population.

With advances in the understanding of steps leading to liver cancer worldwide, researchers have proposed novel treatment strategies, including immunotherapy and targeted therapy [5]. The constant development of novel techniques and drugs provides hope for further advances [6]. The survival rates of patients with liver cancer have improved in certain countries [7]. The Oncology Branch of the Chinese Medical Association revised and updated existing guidelines and issued the “Guidelines for diagnosis and treatment of primary liver cancer in China” to further standardize the diagnosis and treatment [8]. In the recent two decades, China's overall medical and health services have been significantly improved. The difference between the developed and underdeveloped regions has decreased. However, regional differences still clearly exist among different regions across the nation, with the overall pattern being that the medical resources are distributed more in the East than in the West. Potential unevenness of knowledge, therapeutic concepts, and economic status across different regions, hospitals, and individual doctors may exist in the Chinese society, which could create difficulties in the treatment of liver cancer. However, there are no reports on the actual status of liver cancer treatment in mainland China. Therefore, we aimed to conduct this national, multicenter study to investigate the present status of and obstacles in liver cancer treatment especially for advanced-stage liver cancer.

## Methods

### Study population

The study was conducted using a convenience sample. All participants were doctors who had registered on the Beijing iGandan Foundation’s online platform (http://www.gdxz.org.cn/). This is an online academic platform of public welfare focused on hepatobiliary diseases, and has more than 32,000 active users who are doctors from approximately 5000 hospitals across mainland China. One thousand and five hundred doctors from all the 31 provinces in mainland China were randomly selected to be invited through e-mail. These were mainly physicians specializing in liver disease-related diseases, from departments such as the liver disease and infection, at hospitals that were county-level and above. Each participant signed the informed consent at the beginning of the study. Participants were free to withdraw at any stage of the survey, and privacy and confidentiality were ensured.

### Questionnaire design

The questionnaire consisted of three sections (Additional file 1: Table S1). The first section collected general information about the respondents, such as their sex, age, region, professional rank, and hospitals (Table 1). The second section explored the current status of the diagnosis and treatment for liver cancer in China from the following three dimensions: different economic regions, levels of hospitals, and doctors’ professional ranks. Additional file 1: Figure S1 illustrated the economic regions, hospital levels, and doctors' professional ranks in mainland China. According to the National Bureau of Statistics, China is divided into four major economic regions based on geographical location and economic development: East, Central, Western, and Northeast China. The East is the most economically developed with adequate medical resources, while most of the West is economically underdeveloped. Hospitals in mainland China are graded into primary, secondary, and tertiary hospitals. The primary hospitals provide primary health care in communities; secondary hospitals provide medical services across several communities; tertiary hospitals are cross-regional hospitals with comprehensive medical, teaching, and scientific research capabilities. Hospitals of each level are further divided into first, second, and third-class, among which tertiary first-class hospitals possess the highest qualification. Doctors of all professional ranks participated in the study, including residents, attending physicians, deputy chief physicians, and chief physicians. We analyzed the treatment conditions (hospitals’ ability to conduct further tests to determine the nature of the liver masses, the price and adequacy of drugs, and the percentage of patients with advanced cancer encountered in the doctors’ practice), medications [preferred immunotherapy and targeted drugs, methods to deal with adverse effects of targeted therapy, and attitude towards traditional Chinese medicine (TCM)], and treatment strategies (major considerations for prescribing, disclosure of the patients’ true conditions, recommended hospitals for vague diagnosis, and individuals who will make the final decisions on treatment) for liver cancer. The last section comprised multiple-choice questions to collect information on expectations for future treatment. Questions during the survey were asked in Chinese. The questionnaire was translated into English when drafting this manuscript, and checked by a bilingual editor with a medical background.

**Table 1:** Respondent characteristics

Demographic variables	Number (n = 1021)	Percentage (%)
*Sex*		
Male	588	57.6
Female	433	42.4
*Economic regions*		
East	321	31.4
Central	351	34.4
West	255	25.0
Northeast	94	9.2
*Work experience*		
1–5 years	35	3.4
6–15 years	179	17.5
16–25 years	322	31.5
> 25 years	485	47.5
*Professional ranks*		
Resident	27	2.6
Attending physician	136	13.3
Deputy chief physician	356	34.9
Chief physician	502	49.2
*Hospital levels*		
Secondary and lower hospital	265	26.0
Tertiary hospital	185	18.1
Tertiary first-class hospital	571	55.9

### Data collection

The questionnaire was pre-tested by 50 doctors from the Beijing You'an Hospital, and 35 valid questionnaires were recovered. The pre-test did not identify any questions that were inconsistent with the objectives of the questionnaire, questions with wrong options or answers, or questions that most respondents did not answer. The average time to complete the questionnaire was 4 min and 22 s, and the longest was 6 min and 19 s.

A total of 1500 questionnaires were sent out through e-mail, and the participants could answer the questionnaire at their convenience. Considering that doctors from the same hospital may have similar opinions on treatment methods and strategies, especially treatment conditions, a maximum of six doctors from one single hospital were allowed so that the overall outcomes were not influenced by samples from a few hospitals with too many invited participants. If more than six questionnaires were collected from the same hospital, only six of them were randomly selected and the extra ones were regarded as invalid and thus excluded from subsequent analysis. A few quite easy questions irrelevant to the topic (for example, arithmetic problems) were set as quality control. A tiny minority of participants who answered incorrectly to such questions were considered as distracted or not serious during the survey, and thus their questionnaires were regarded as invalid. We excluded questionnaires that took more than 10 min to complete to ensure that no participant looked up references while filling the questionnaire. Finally, 1021 valid questionnaires from 821 hospitals were collected, with an effective response rate of 68%.

### Data analysis

We used IBM SPSS Statistics for Windows, version 26.0, (IBM Corp, Armonk, NY) to process the research data. The variables are presented as frequency and ratio. To study the current status of liver cancer treatment, the participants were grouped according to their region, professional rank, and level of hospital. We performed chi-square and logistic regression analyses to explore the differences caused by the above-mentioned factors. To investigate the barriers in liver cancer treatment, we grouped the doctors according to their satisfaction with the current treatment. The positive results of the univariate analysis and other potential influencing factors were included in the multivariate logistic regression analysis to identify obstacles in the treatment of liver cancer. For all tests and analyses, a *P*-value < 0.05 was considered statistically significant.

## Result

### Participant characteristics

As shown in Table 1, a total of 1021 participants were finally included in the study, among whom 588 (57.6%) were male. There were 321 (31.4%), 351 (34.4%), 255 (25.0%), and 94 (9.2%) participants coming from East, Central, West, and Northeast China, respectively. Approximately half (55.9%) of the participants worked in tertiary first-class hospitals, less than twenty percent (18.1%) worked in tertiary hospitals, and the others (26.0%) worked in secondary and lower level hospitals. There were 502 (49.2%) chief physicians, 356 (34.9%) deputy chief physicians, 136 (13.3%) attending physicians, and 27 (2.6%) residents. The majority had medical practice of more than 5 years.

### Treatment status

#### Differences in liver cancer treatment among different regions with different economic status

Some statistically significant differences among different economic regions were found, including hospitals' ability to confirm the nature of liver masses (*P* = 0.004, Table 2), preferred choice of targeted drugs (*P* < 0.001, Table 2), and major considerations for prescribing medications (*P* = 0.008, Table 2). Notably, correlations were further investigated using logistic analysis.

**Table 2:** Differences in the treatment conditions, medications, and treatment strategies among different economic regions

Questions	Answers	East China (n = 321)	Central China (n = 351)	West China (n = 255)	Northeast China (n = 94)	*P*-value
*Treatment conditions*						
**Is the price of medication acceptable?**	**Yes**	**112 (34.9%)**	**84 (23.9%)**	**67 (26.3%)**	**24 (25.5%)**	0.011*
	**No**	**209 (65.1%)**	**267 (76.1%)**	**188 (73.7%)**	**70 (74.5%)**	
Are drugs sufficient?	Yes	73 (22.7%)	87 (24.8%)	57 (22.4%)	33 (35.1%)	0.075
	No	248 (77.3%)	264 (75.2%)	198 (77.6%)	61 (64.9%)	
**What is your major drug source?**	**Imported**	**89 (27.7%)**	**118 (33.6%)**	**88 (34.5%)**	**43 (45.7%)**	0.011*
	**Domestic**	**188 (58.6%)**	**169 (48.1%)**	**132 (51.8%)**	**40 (42.6%)**	
	**Available**	**44 (13.7%)**	**64 (18.2%)**	**35 (13.7%)**	**11 (11.7%)**	
**What is the percentage of the advanced stage cancer in your practice?**	** < 40%**	**100 (31.2%)**	**85 (24.2%)**	**76 (29.8%)**	**39 (41.5%)**	0.012*
	**40–60%**	**129 (40.2%)**	**134 (38.2%)**	**90 (35.3%)**	**33 (35.1%)**	
	** > 60%**	**92 (28.7%)**	**132 (37.6%)**	**89 (34.9%)**	**22 (23.4%)**	
What is the percentage of the first diagnosis?	< 20%	163 (50.8%)	183 (52.1%)	142 (55.7%)	52 (55.3%)	0.621
	20–40%	94 (29.3%)	109 (31.1%)	66 (25.9%)	22 (23.4%)	
	> 40%	64 (19.9%)	59 (16.8%)	47 (18.4%)	20 (21.3%)	
Is the method of diagnosis enough?	Yes	166 (51.7%)	183 (52.1%)	117 (45.9%)	47 (50.0%)	0.436
	No	155 (48.3%)	168 (47.9%)	138 (54.1%)	47 (50.0%)	
**Is your hospital able to conduct further tests to determine the nature of the liver masses?**	**Yes**	**312 (97.2%)**	**341 (97.2%)**	**244 (95.7%)**	**84 (89.4%)**	0.004*
	**No**	**9 (2.8%)**	**10 (2.8%)**	**11 (4.3%)**	**10 (10.6%)**	
*Medication methods*						
**Do you advocate traditional Chinese medicine?**	**Yes**	**194 (60.4%)**	**243 (69.2%)**	**146 (57.3%)**	**51 (54.3%)**	0.005*
	**No**	**127 (39.6%)**	**108 (30.8%)**	**109 (42.7%)**	**43 (45.7%)**	
What is your preferred immunotherapy drug?	PD-1	246 (76.6%)	273 (77.8%)	179 (70.2%)	75 (79.8%)	0.054
	PD-L1	70 (21.8%)	62 (17.7%)	62 (24.3%)	16 (17.0%)	
	CTLA-4	5 (1.6%)	16 (4.6%)	14 (5.5%)	3 (3.2%)	
How do you deal with adverse effects of targeted therapy?	Keeping the dosage and frequency	108 (33.6%)	116 (33.0%)	97 (38.0%)	35 (37.2%)	0.844
	Reducing the dosage	174 (54.2%)	186 (53.0%)	125 (49.0%)	48 (51.1%)	
	Intermittent medication	39 (12.1%)	49 (14.0%)	33 (12.9%)	11 (11.7%)	
**What is your preferred targeted drug?**	**Sorafenib**	**151 (47.0%)**	**200 (57.0%)**	**162 (63.5%)**	**59 (62.8%)**	<0.001*
	**Lenvatinib**	**145 (45.2%)**	**109 (31.1%)**	**65 (25.5%)**	**25 (26.6%)**	
	**Second-line drugs**	**25 (7.8%)**	**42 (12.0%)**	**28 (11.0%)**	**10 (10.6%)**	
*Treatment strategies*						
**What are your major considerations for prescribing?**	**Cost or insurance**	**95 (29.6%)**	**127 (36.2%)**	**97 (38.0%)**	**41 (43.6%)**	0.008*
	**Effectiveness**	**174 (54.2%)**	**174 (49.6%)**	**106 (41.6%)**	**40 (42.6%)**	
	**Availability**	**52 (16.2%)**	**50 (14.2%)**	**52 (20.4%)**	**13 (13.8%)**	
What is your preferred treatment regimen?	Targeted therapy	36 (11.2%)	39 (11.1%)	28 (11.0%)	11 (11.7%)	0.962
	Immunotherapy	5 (1.6%)	4 (1.1%)	3 (1.2%)	0 (0.0%)	
	Target therapy & Immunotherapy	272 (84.7%)	301 (85.8%)	217 (85.1%)	81 (86.2%)	
	Chemotherapy	8 (2.5%)	7 (2.0%)	7 (2.7%)	2 (2.1%)	
**Do you support the disclosure of the patients’ true conditions?**	**Yes**	**225 (70.1%)**	**205 (58.4%)**	**147 (57.6%)**	**55 (58.5%)**	0.004*
	**No**	**96 (29.9%)**	**146 (41.6%)**	**108 (42.4%)**	**39 (41.5%)**	
Who will make the final decisions on treatment?	Doctors	37 (11.5%)	40 (11.4%)	46 (18.0%)	18 (19.1%)	0.113
	Patients	75 (23.4%)	88 (25.1%)	61 (23.9%)	18 (19.1%)	
	Patients’ family	209 (65.1%)	223 (63.5%)	148 (58.0%)	58 (61.7%)	
**Is the pathological diagnosis important?**	**Yes**	**159 (49.5%)**	**172 (49.0%)**	**148 (58.0%)**	**40 (42.6%)**	0.036*
	**No**	**162 (50.5%)**	**179 (51.0%)**	**107 (42.0%)**	**54 (57.4%)**	
**What is your recommended hospital when the diagnosis is vague?**	**Local hospitals**	**83 (25.9%)**	**31 (8.8%)**	**23 (9.0%)**	**16 (17.0%)**	<0.001*
	**Provincial capital hospitals**	**86 (26.8%)**	**206 (58.7%)**	**136 (53.3%)**	**34 (36.2%)**	
	**National top hospitals**	**152 (47.4%)**	**114 (32.5%)**	**96 (37.6%)**	**44 (46.8%)**	

Bold and * indicate statistical significance (*P* < 0.05)

In terms of treatment conditions, the hospitals’ ability to conduct further tests to determine the nature of the liver masses was better in East China than in Northeast China (odds ratio, OR 4.127, 95% CI 1.625–10.483 *P* = 0.003). Compared with those in East China, fewer doctors in Central and West China considered the drugs inexpensive (OR 0.587 for Central China, 95% CI 0.420–0.821, *P* = 0.002; OR 0.665 for West China, 95% CI 0.463–0.954, *P* = 0.027). In addition, doctors in Central China encountered higher percentage of the advanced stage cancer than those in East China (OR 1.688, 95% CI 1.139–2.501, *P* = 0.009).

Regarding the medications for liver cancer, considering sorafenib a reference, participants in other regions were less inclined to choose lenvatinib than those in East China. (OR 0.568 for Central China, 95% CI 0.410–0.786, *P* = 0.001; OR 0.418 for West China, 95% CI 0.289–0.603, *P* < 0.001; and OR 0.441 for Northeast China, 95% CI 0.262–0.742, *P* = 0.002). TCM was less likely to be recommended in East China than in Central China (OR 0.679, 95% CI 0.494–0.934, *P* = 0.017).

With regard to treatment strategies, recommendations to provincial capital hospitals were more preferred in other regions than in East China (OR 6.413 for Central China *P* < 0.001; 95% CI 3.956–10.398, OR 5.707 for West China, 95% CI 3.342–9.744, *P* < 0.001; and OR 2.051 for Northeast China, 95% CI 1.053–3.993, *P* = 0.035). The recommendation for treatment in national top hospitals across the country was more preferred in Central and West China than in East China (OR 2.008 for Central China, 95% CI 1.244–3.241, *P* = 0.004; OR 2.279 for West China, 95% CI 1.344–3.864, *P* = 0.002). Doctors in East China prioritized pathological examinations less than those in West China (OR 0.710, 95% CI 0.509–0.988, *P* = 0.042). Informing patients of their true conditions was less supported in other regions than in East China (OR 0.599 for Central China, 95% CI 0.435–0.825, *P* = 0.002; OR 0.581 for West China, 95% CI 0.411–0.820, *P* = 0.002; OR 0.602 for Northeast China, 95% CI 0.374–0.967, *P* = 0.036). In addition, doctors in West and Northeast China paid more attention to cost or insurance than those in East China while prescribing drugs. Instead, they considered curative effects less important (OR 0.597 for West China, 95% CI 0.411–0.865, *P* = 0.007; OR 0.533 for Northeast China, 95% CI 0.322–0.880, *P* = 0.014).

#### Differences in liver cancer treatment among different levels of hospitals

Different levels of hospitals displayed some significant differences in the availability of drugs (*P* = 0.038, Table 3), attitudes towards TCM (*P* < 0.001, Table 3), and opinions on the disclosure of true conditions to the patients (*P* < 0.001, Table 3). Related data was presented in Table 3 in detail, and some positive findings were further investigated via logistic analysis.

**Table 3:** Differences in the treatment conditions, medications, and treatment strategies among different levels of hospitals

Questions	Answers	Tertiary first-class hospital (n = 571)	Tertiary hospital (n = 185)	Secondary and lower hospital (n = 265)	*P*-value
*Treatment conditions*					
**Is the price of medication acceptable?**	**Yes**	**398 (69.7%)**	**148 (80.0%)**	**188 (70.9%)**	0.024*
	**No**	**173 (30.3%)**	**37 (20.0%)**	**77 (29.1%)**	
**Are drugs sufficient?**	**Yes**	**146 (25.6%)**	**32 (17.3%)**	**72 (27.2%)**	0.038*
	**No**	**425 (74.4%)**	**153 (82.7%)**	**193 (72.8%)**	
What is your major drug source?	Imported	188(32.9%)	66(35.7%)	84(31.7%)	0.240
	Domestic	306(53.6%)	93(50.3%)	130(49.1%)	
	Available	77(13.5%)	26(14.1%)	51(19.2%)	
What is the percentage of the advanced stage cancer in your practice?	< 40%	165 (28.9%)	48 (25.9%)	87 (32.8%)	0.315
	40–60%	222 (38.9%)	77 (41.6%)	87 (32.8%)	
	> 60%	184 (32.2%)	60 (32.4%)	91 (34.3%)	
What is the percentage of first diagnosis?	< 20%	289 (50.6%)	94 (50.8%)	157 (59.2%)	0.058
	20–40%	161 (28.2%)	58 (31.4%)	72 (27.2%)	
	> 40%	121 (21.2%)	33 (17.8%)	36 (13.6)	
Is the method of diagnosis enough?	Yes	290 (50.8%)	81 (43.8%)	142 (53.6%)	0.114
	No	281 (49.2%)	104 (56.2%)	123 (46.4%)	
**Is your hospital able to conduct further tests to determine the nature of the liver masses?**	**Yes**	**564 (98.8%)**	**180 (97.3%)**	**237 (89.4%)**	<0.001*
	**No**	**7 (1.2%)**	**5 (2.7%)**	**28 (10.6%)**	
*Medication methods*					
**Do you advocate traditional Chinese medicine?**	**Yes**	**330 (57.8%)**	**115 (62.2%)**	**189 (71.3%)**	<0.001*
	**No**	**241 (42.2%)**	**70 (37.8%)**	**76 (28.7%)**	
What is your preferred immunotherapy drug?	PD-1	445 (77.9%)	134 (72.4%)	194 (73.2%)	0.253
	PD-L1	106 (18.6%)	41 (22.2%)	63 (23.8%)	
	CTLA-4	20 (3.5%)	10 (5.4%)	8 (3.0%)	
**How do you deal with adverse effects of targeted therapy?**	**Keeping the dosage and frequency**	**197(34.5%)**	**72(38.9%)**	**87(32.8%)**	0.020*
	**Reducing the dosage**	**307(53.8%)**	**97(52.4%)**	**129(48.7%)**	
	**Intermittent medication**	**67(11.7%)**	**16(8.6%)**	**49(18.5%)**	
**What is your preferred targeted drug?**	**Sorafenib**	**326 (57.1%)**	**102 (55.1%)**	**144 (54.3%)**	<0.001*
	**Lenvatinib**	**209 (36.6%)**	**56 (30.3%)**	**79 (29.8%)**	
	**Second-line drugs**	**36 (6.3%)**	**27 (14.6%)**	**42 (15.8%)**	
*Treatment strategies*					
What are your major considerations for prescribing?	Cost or insurance	199 (34.9%)	68 (36.8%)	93 (35.1%)	0.766
	Effectiveness	285 (49.9%)	84 (45.4%)	125(47.2%)	
	Availability	87(15.2%)	33(17.8%)	47(17.7%)	
What is your preferred treatment regimen?	Targeted therapy	58(10.2%)	20(10.8%)	36(13.6%)	0.400
	Immunotherapy	8(1.4%)	3(1.6%)	1(0.4%)	
	Target therapy & Immunotherapy	489(85.6%)	157(84.9%)	225(84.9%)	
	Chemotherapy	16(2.8%)	5(2.7%)	3(1.1%)	
**Do you support the disclosure of the patients’ true conditions?**	**Yes**	**382(66.9%)**	**110(59.5%)**	**140(52.8%)**	<0.001*
	**No**	**189(33.1%)**	**75(40.5%)**	**125(47.2%)**	
Who will make the final decisions on treatment?	Doctors	84(14.7%)	22(11.9%)	35(13.2%)	0.555
	Patients	129(22.6%)	52(28.1%)	61(23.0%)	
	Patients’ family	358(62.7%)	111(60.0%)	169(63.8%)	
Is the pathological diagnosis important?	Yes	285(49.9%)	100(54.1%)	134(50.6%)	0.616
	No	286(50.1%)	85(45.9%)	131(49.4%)	
**What is your recommended hospital when the diagnosis is vague?**	**Local hospitals**	**65 (11.4%)**	**27 (14.6%)**	**61 (23.0%)**	<0.001*
	**Provincial capital hospitals**	**199 (34.9%)**	**109 (58.9%)**	**154 (58.1%)**	
	**National top hospitals**	**307 (53.8%)**	**49 (26.5%)**	**50 (18.9%)**	

Bold and * indicate statistical significance (*P* < 0.05)

With regard to treatment conditions, the drugs were considered insufficient (OR 1.642, 95% CI 1.074–2.512, *P* = 0.022) as well as costly (OR 0.575, 95% CI 0.385–0.860, *P* = 0.007) in tertiary hospitals compared with tertiary first-class hospitals.

Regarding medications, upon encountering adverse effects of the targeted therapy, doctors in secondary and lower hospitals tended to recommend intermittent medications, whereas maintaining the dosage and frequency was more likely to be suggested in tertiary first-class hospitals (OR 1.656, 95% CI 1.060–2.588, *P* = 0.027). Doctors in tertiary first-class hospitals valued TCM less than those in secondary and lower hospitals (OR 0.551, 95% CI 0.402–0.754, *P* < 0.001).

Regarding treatment strategies, informing patients of their true conditions was more approved in tertiary first-class hospitals than in secondary and lower hospitals (OR 1.805, 95% CI 1.340–2.430, *P* < 0.001). Compared with recommending patients to other local hospitals, doctors in lower-level hospitals were less inclined to recommend patients to national top hospitals nationwide (OR 0.384 for tertiary hospitals, 95% CI 0.224–0.660, *P* = 0.001; OR 0.174 for secondary and lower hospitals, 95% CI 0.110–0.275, *P* < 0.001).

#### Differences in liver cancer treatment among doctors of different professional ranks

Doctors of different professional ranks showed some significant differences, including the major source of drugs (*P* = 0.048, Table 4), hospital’ ability to conduct further tests (*P* = 0.003, Table 4), preferred choice of immunotherapy drugs (*P* = 0.011, Table 4), and opinions on disclosure of the true conditions to patients (*P* < 0.001, Table 4). Detail information was present in Table 4, and logistic analysis was utilized to investigate some interesting findings further.

**Table 4:** Differences in the treatment conditions, medications, and treatment strategies among doctors of different professional ranks

Questions	Answers	Chief physicians (n = 502)	Deputy chief physicians (n = 356)	Attending physicians and residents (n = 163)	*P*-value
*Treatment conditions*					
Is the price of medication acceptable?	Yes	353 (70.3%)	258 (72.5%)	123 (75.5%)	0.427
	No	149 (29.7%)	98 (27.5%)	40 (24.5%)	
Are drugs sufficient?	Yes	133 (26.5%)	79 (22.2%)	38 (23.3)	0.328
	No	369 (73.5%)	277 (77.8%)	125 (76.7%)	
**What is your major drug source?**	**Imported**	**156 (31.1%)**	**132 (37.1%)**	**50 (30.7%)**	0.048*
	**Domestic**	**274 (54.6%)**	**177 (49.7%)**	**78 (47.9%)**	
	**Available**	**72 (14.3%)**	**47 (13.2%)**	**35 (21.5%)**	
What is the percentage of the advanced stage cancer in your practice?	< 40%	153 (30.5%)	91 (25.6%)	56 (34.4%)	0.226
	40–60%	184 (36.7%)	140 (39.3%)	62 (38.0%)	
	> 60%	165 (32.9%)	125 (35.1%)	45 (27.6%)	
What is the percentage of the first diagnosis?	< 20%	265 (52.6%)	182 (51.1%)	94 (57.7%)	0.157
	20–40%	134 (26.7%)	116 (32.6%)	41 (25.2%)	
	> 40%	104 (20.7%)	58 (16.3%)	28 (17.2%)	
Is the method of diagnosis enough?	Yes	239(47.6%)	180(50.6%)	94(57.7%)	0.082
	No	263(52.4%)	176(49.4%)	69(42.3%)	
**Is your hospital able to conduct further tests to determine the nature of the liver masses?**	**Yes**	**488(97.2%)**	**344(96.6%)**	**149(91.4%)**	0.003*
	**No**	**14(2.8%)**	**12(3.4%)**	**14(8.6%)**	
*Medication methods*					
Do you advocate traditional Chinese medicine?	Yes	314 (62.5%)	217 (61.0%)	103 (63.2%)	0.851
	No	188 (37.5%)	139 (39.0%)	60 (36.8%)	
**What is your preferred immunotherapy drug?**	**PD-1**	**383 (76.3%)**	**279 (78.4%)**	**111 (68.1%)**	0.011*
	**PD-L1**	**100 (19.9%)**	**61 (17.1%)**	**49 (30.1%)**	
	**CTLA-4**	**19 (3.8%)**	**16 (4.5%)**	**3 (1.8%)**	
How do you deal with adverse effects of targeted therapy?	Keeping the dosage and frequency	177 (35.3%)	124 (34.8%)	55 (33.7%)	0.688
	Reducing the dosage	268 (53.4%)	181 (50.8%)	84 (51.5%)	
	Intermittent medication	57 (11.4%)	51 (14.3%)	24 (14.7%)	
What is your preferred targeted drug?	Sorafenib	275 (54.8%)	205 (57.6%)	92 (56.4%)	0.302
	Lenvatinib	182 (36.3%)	113 (31.7%)	49 (30.1%)	
	Second-line drugs	45 (9.0%)	38 (10.7%)	22 (13.5%)	
*Treatment strategies*					
What are your major considerations for prescribing?	Cost or insurance	178 (35.5%)	126 (35.4%)	56 (34.4%)	0.794
	Effectiveness	249 (49.6%)	166 (46.6%)	79 (48.5%)	
	Availability	75 (14.9%)	64 (18.0%)	28 (17.2%)	
What is your preferred treatment regimen?	Targeted therapy	63(12.5%)	31(8.7%)	20(12.3%)	0.264
	Immunotherapy	5(1.0%)	3(0.8%)	4(2.5%)	
	Target therapy & Immunotherapy	421(83.9%)	316(88.8%)	134(82.2%)	
	Chemotherapy	13(2.6%)	6(1.7%)	5(3.1%)	
**Do you support the disclosure of the patients’ true conditions?**	**Yes**	**336(66.9%)**	**215(60.4%)**	**81(49.7%)**	<0.001*
	**No**	**166(33.1%)**	**141(39.6%)**	**82(50.3%)**	
**Who will make the final decisions on treatment?**	**Doctors**	**89(17.7%)**	**39(11.0%)**	**13(8.0%)**	0.004*
	**Patients**	**121(24.1%)**	**86(24.2)**	**35(21.5%)**	
	**Patients’ family**	**292(58.2%)**	**231(64.9%)**	**115(70.6%)**	
**Is the pathological diagnosis important?**	**Yes**	**230(45.8%)**	**191(53.7%)**	**98(60.1%)**	0.003*
	**No**	**272(54.2%)**	**165(46.3%)**	**65(39.9%)**	
**What is your recommended hospital when the diagnosis is vague?**	**Local hospitals**	**61(12.2%)**	**64(18.0%)**	**28(17.2%)**	<0.001*
	**Provincial capital hospitals**	**197(39.2%)**	**170(47.8%)**	**95(58.3%)**	
	**National top hospitals**	**244(48.6%)**	**122(34.3%)**	**40(24.5%)**	

Bold and * indicate statistical significance (*P* < 0.05)

Regarding the medications, programmed death-ligand 1 (PD-L1) was more likely to be opted for immunotherapy by the attending physicians and residents than by chief physicians, than programmed cell death protein 1 (PD-1) (OR 1.691, 95% CI 1.131–2.527, *P* = 0.010).

In terms of treatment strategies, doctors of higher professional ranks preferred informing patients of their true conditions (OR 0.753 for deputy chief physicians, 95% CI 0.568–0.999, *P* = 0.049; OR 0.488 for attending physicians and residents, 95% CI 0.341–0.699, *P* < 0.001). The pathological diagnosis was prioritized more by those of lower professional ranks (OR 0.561 for deputy chief physicians, 95% CI 0.392–0.803, *P* = 0.002; OR 0.730 for attending physicians and residents, 95% CI 0.556–0.959, *P* = 0.024). Chief physicians tended to select treatment solutions for the patients, whereas deputy chief physicians tended to let the patients select the treatment plan by themselves (OR 1.622, 95% CI 1.017–2.587, *P* = 0.042).

### Obstacles in treatment

Physicians involved in this study were divided into two groups according to their response to the question regarding whether the current treatment for liver cancer was satisfactory. Among them, only 23.4% of doctors were satisfied with the current treatment effect of liver cancer, and the remaining 76.6% of doctors were dissatisfied. The percentage of doctors who were optimistic about the treatment varied across different regions (*P* = 0.015). Doctors who reported a high percentage of patients with advanced-stage liver cancer were more likely to have negative attitudes towards current treatment options for liver cancer (*P* < 0.001). The unsatisfied group was also correlated with pessimistic attitudes towards current diagnostic methods and medicine being sufficient for clinical needs (both *P* < 0.001). The doctors of both groups demonstrated significantly different methods to deal with adverse effects caused by the targeted drugs (*P* = 0.003), with more doctors in the satisfied group displaying a tendency to maintain the therapeutic dosage. In addition, those who supported informing the patients of their true conditions tended to be satisfied with the current treatment (*P* = 0.032).

Univariate logistic analysis was conducted to find the correlation between doctors’ satisfaction with the current treatment and other attitudes or considerations (Additional file 1: Table S2). Those statistically significant correlations were included in the multivariate analysis for further investigation. Moreover, we included the doctors’ professional ranks (*P* = 0.696), hospital levels (*P* = 0.240), and preferred choice of targeted drugs (*P* = 0.065), as factors that potentially influenced doctors’ attitude towards the current treatment, despite not reaching statistical significance in the univariate analysis.

**Fig. 1:**
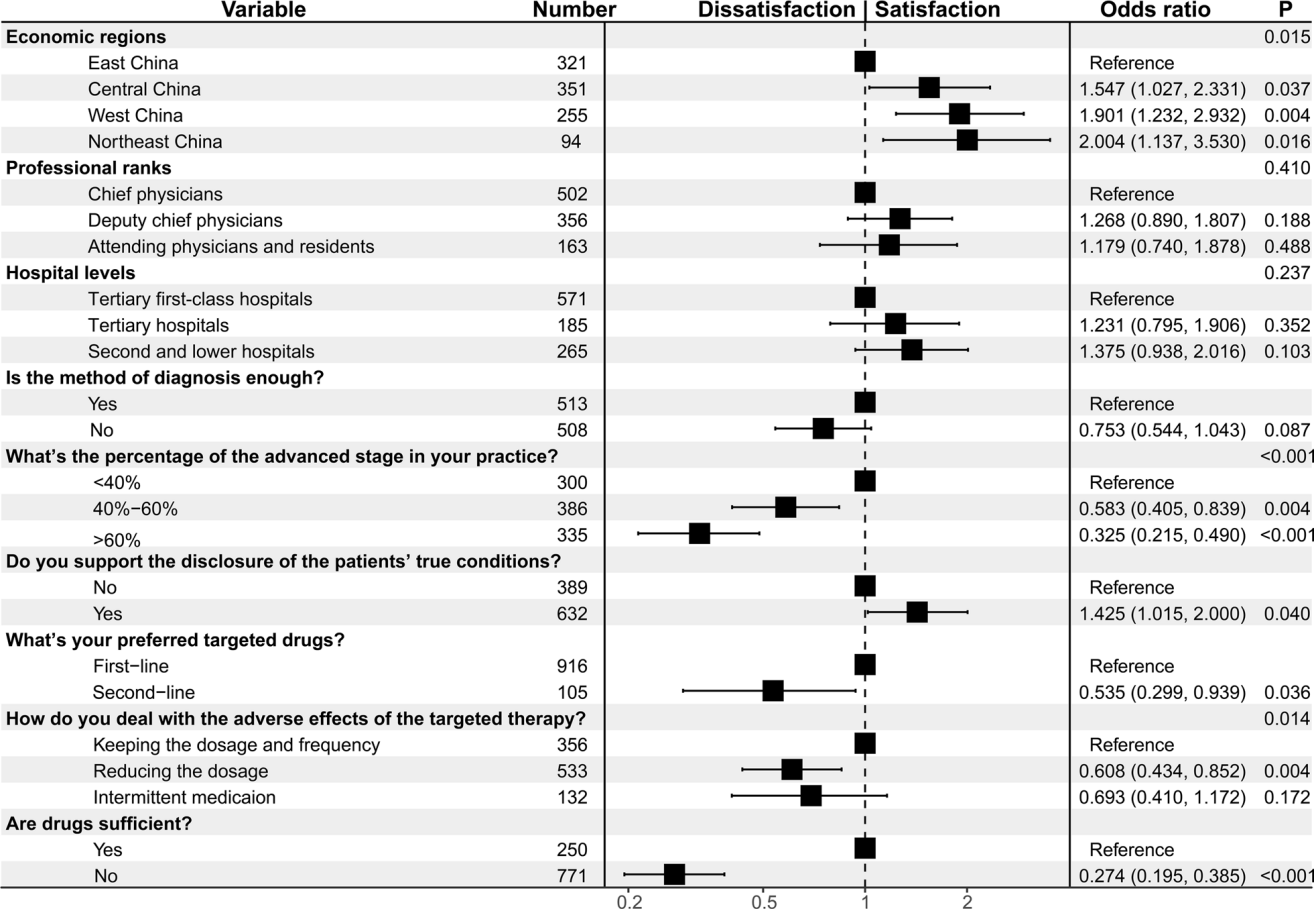
Differences in baseline demographics and treatments between satisfied and dissatisfied physicians

As shown in Fig. 1, the economic regions (*P* = 0.015), percentages of advanced-stage liver cancer in the doctors’ practice (*P* < 0.001), preferred choice of targeted drugs (*P* = 0.036), methods to deal with adverse effects of targeted drugs (*P* = 0.014), informing patients of their real conditions (*P* = 0.040), and attitudes towards the current medicine (*P* < 0.001) were independently associated with the satisfaction of doctors with the current treatment for liver cancer, whereas the doctors’ professional ranks (*P* = 0.410), hospital levels (*P* = 0.237), and attitude towards the methods of diagnosis (*P* = 0.087) failed to display a correlation (Fig. 1). Compared with those from East China, doctors from other regions tended to be satisfied with the current treatment (OR 1.548 for Middle China, *P* = 0.037; OR 1.901 for West China, and *P* = 0.004; OR 2.004 for Northeast China, *P* = 0.016). A higher percentage of advanced-stage liver cancer patients in the doctors’ practice suggested that their doctors were less likely to be satisfied (< 40% as the reference, OR 0.583 for 40–60%, *P* = 0.004; OR 0.325 for > 60%, *P* < 0.001). A pessimistic attitude towards the current medicine was negatively related to their satisfaction with the treatment for liver cancer (OR 0.274, *P* < 0.001). The preferred choice of second-line targeted drugs rather sorafenib and lenvatinib impaired the satisfaction of doctors (OR 0.535, *P* = 0.036). Regarding the methods to deal with adverse effects caused by the targeted drugs, dosage reduction displayed a negative correlation with doctors’ satisfaction (OR 0.608, *P* = 0.004) than dosage maintenance. However, there was no significant difference in intermittent medications (OR 0.693, *P* = 0.172). Interestingly, the willingness to inform patients of their true conditions was a protective indicator of the optimistic attitude towards the current treatment for liver cancer (OR 1.425, *P* = 0.040).

### Future expectations of doctors and patients

**Fig. 2:**
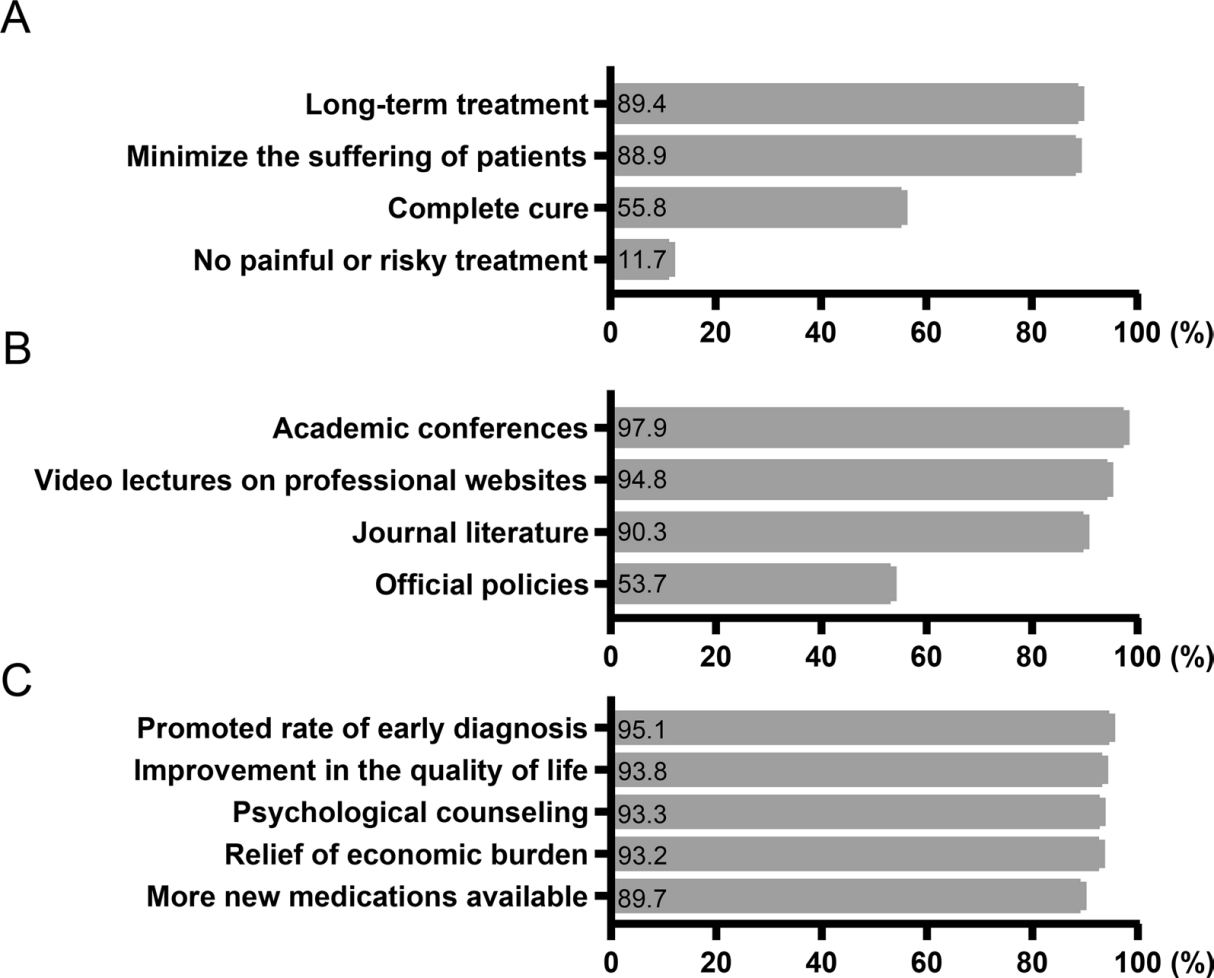
Future expectations of doctors and patients. **A** Which of the following treatment models do you expect for liver cancer? **B** How would you like to get access to the updated knowledge on the progress of liver cancer diagnosis and treatment? **C** What do patients with liver cancer need as far as you are concerned?

Nearly 90% of the doctors agreed with two treatment modes for liver cancer, as follows: (1) long-term treatment, in which the patients lived with tumors and the disease progressed slowly; (2) to minimize the suffering of patients and improve their quality of lives. However, approximately half of the doctors hoped for a complete cure, and only one-fifth of the doctors agreed on not implementing any painful and risky treatment (Fig. 2A). More than 90% of the doctors displayed strong expectations for obtaining updated knowledge about the progress of liver cancer diagnosis and treatment by participating in academic conferences, accessing journal literature, and attending video lectures on professional websites (Fig. 2B). Moreover, approximately 90% of the doctors believed that patients with liver cancer were in demand of increased early diagnosis rate, more novel medications, the relief of economic burden, an improvement in the quality of life, and psychological counseling (Fig. 2C).

## Discussion

This article used 1021 questionnaires to analyze the current status of and obstacles in the treatment of liver cancer treatment in China. We identified differences in the treatment of liver cancer in China, particularly across diverse economic regions. While most doctors are unhappy with the present treatment for liver cancer, early diagnosis and the revelation of true conditions to patients were recognized as the contributing factors.

In 2015, the incidence of liver cancer in the economically developed eastern region of China was 24.46/100,000, compared with 27.41/100,000 and 29.56/100,000 in the relatively underdeveloped central and western regions, respectively. Liver cancer-related mortality was highest in the western region, at 2.545/100,000, followed by 24.18/100,000 and 21.98/100,000 in the central and eastern regions, respectively [9]. High mortality due to liver cancer in economically undeveloped areas was closely related to its high incidence, and relatively incomplete treatment conditions for liver cancer were likely an important influencing factor. With a non-negligible differences in the distribution of health resources across different regions of China, which is a developing country with economic diversity [10, 11], major cancer treatments and health service use were more concentrated in rich patients than in poor patients [12]. In the present survey, we reported on significant differences in treatment conditions for liver cancer among different regions. Hospitals’ ability to determine the nature of the liver masses, drug price, and tumor staging were more positive in economically developed regions. In addition, doctors paid more attention to cost or insurance, which were realistic considerations in the treatment for liver cancer, compared with curative effects, in less developed regions. Therefore, more attention should be paid to alleviating the differences in the treatment of liver cancer among different regions in China.

The vague symptoms of liver cancer at an early stage lead to the diagnosis at an advanced stage in most patients, which excludes the possibility of local treatments, such as curative hepatic resection, tumor ablation, or trans-arterial therapy. Therefore, the systemic treatment of advanced liver cancer has attracted much attention. The current first-line treatment includes sorafenib, introduced in 2007 [13], and lenvatinib [14], introduced in 2017. Furthermore, second-line treatments, such as regorafenib and cabozantinib [14], are available. In addition, PD-1 and PD-L1 are available for liver cancer immunotherapy [15]. With remarkable advancements in targeted therapy and immunotherapy for advanced liver cancer, selecting the most suitable medication for patients has become a new concern. Most doctors preferred lenvatinib, particularly in tertiary first-class hospitals. A phase III randomized, multicenter, open-label, non-inferiority trial on first-line targeted therapy drugs demonstrated that lenvatinib achieved better overall survival benefits and longer median progression-free survival than sorafenib [16]. Moreover, lenvatinib is less costly than sorafenib. In this study, lenvatinib was more preferred in East China than in other regions, compared with the conventional first-line targeted therapy drug sorafenib. This may be attributed to limited availability of updated knowledge about liver cancer treatment to doctors in Central, West, and Northeast China than to those in East China.

Targeted therapies, such as sorafenib, lenvatinib, regulafenib, and cabozantinib, are associated with adverse events that negatively impact the quality of life of patients [17]. Severe drug resistance to targeted therapy is maintained during long-term application [18]. Low-cost TCM can be used for comprehensive treatment with fewer adverse events and multitarget regulation characteristics. In the early stages of tumor development, TCM can be used as a therapeutic regimen to alleviate protumor factors. Meanwhile, it can work as an adjuvant therapy to improve the survival, alleviate drug-related adverse effects, and improve quality of life in the intermediate and terminal stages. Therefore, the application of TCM has been advocated in different stages of liver cancer development [19]. As reported by the World Health Organization, traditional medicines principally derived from plants serve as a fundamental part of the primary health care of the major population in developing countries [20]. China is the origin of TCM and is the largest developing country with a population of 1.4 billion. In this study, two-third of the doctors approved the use of TCM in the treatment of liver cancer. However, clinicians of tertiary first-class hospitals, which were the highest-ranked hospitals, were inclined to disapprove of the application of TCM. This was possibly attributed to the availability of adequate medications to doctors from tertiary first-class hospitals.

Informing patients themselves with cancer about the exact diagnoses and poor prognoses remains debatable, without a consensus on this issue [21]. In the clinical setting, undoubtedly it is crucial to safeguard the patients’ autonomy and right to be informed. Yet, in some circumstances, things can be a little different: when patients are diagnosed with diseases with dismal prognoses, their relatives may show kindness by concealing the truth to comfort and encourage them. In China, where family harmony is perceived as one of the most vital social values, doctors adopt a family‐centered approach to cancer diagnoses/prognoses disclosure. If family members decide not to disclose the diagnosis/prognosis, doctors will honor this decision and conceal the diagnosis/prognosis from the patient. In this study, we investigated whether the doctors agreed to disclose the true conditions of liver cancer to patients. Sixty percent of the doctors believed that patients with liver cancer should be informed of their true condition. Further analysis demonstrated that telling the truth was more supported by doctors who were satisfied with the treatment results. In addition, doctors from economically developed areas and high-level hospitals and those of higher professional ranks were more likely to disclose the true conditions. Furthermore, the negative consequences of non-disclosure are not limited to the neglect of patients' natural rights. Poor physical conditions [22], increased mental illnesses, and decreased trust in family members and doctors [23] have been reported in patients during the process of seeking out the truth. Thus, disclosing the truth about the diagnosis and prognosis of patients seems beneficial. Nonetheless, further research is warranted to confirm the internal link between disclosure and the treatment for liver cancer.

The prognosis of liver cancer is considerably driven by the tumor stage [24]. The 5-year survival rate in patients detected at an early stage approaches 70% [25], whereas those with advanced tumors have much poorer prognoses, with a median survival of 1–2 years [26]. A higher percentage of advanced-stage cancer in the doctors’ practice suggested that the doctors were less likely to be satisfied with the current treatment of liver cancer. The implementation of primary preventive measures for liver cancer in China is an important way to reduce its disease burden; however, clinicians should consider its secondary prevention, which involves detecting the lesions timely, thus improving the treatment efficacy and reducing the mortality [9].

In this study, hospital levels and doctors’ professional ranks were not associated with the satisfaction of doctors with the treatment effect. Compared with doctors from East China, those from other regions tended to be satisfied with the current treatment for liver cancer, thereby suggesting possible higher expectations for treatment efficacy in East China.

This study had some limitations. First, this questionnaire-based study was based on a cross-sectional survey, which could not reflect the dynamic changes in the doctors’ perspectives on the treatment of liver cancer. In addition, doctors invited to complete the questionnaire were randomly selected from a convenience sample, which could compromise the representativeness and thus lead to bias. Despite a small proportion of patients being suitable for surgery, we barely discussed local–regional treatment, with the questionnaire focusing on the systemic treatment for liver cancer. However, this nationwide study with a relatively large sample provided valuable references for unraveling the current status of and obstacles in the treatment of liver cancer.

## Conclusion

This is the first questionnaire-based study to investigate the current status of and obstacles in the diagnosis and treatment of liver cancer from doctors’ perspectives in China. Our findings highlighted the need to pay attention to improving the subpar level of detection and treatment for liver cancer in underdeveloped areas. In addition, early diagnosis, disclosure of true conditions to the patients, choice of system therapy drugs, and application of TCM were positively correlated with the doctors’ satisfaction with liver cancer treatment. This necessitates efforts to promote the current status of liver cancer treatment and achieve better medical services for patients with liver cancer in China.

## Supplementary Information

**Additional file 1**. **Supplementary Table 1.** Detailed information of the questionnaire. **Supplementary Table 2.** Correlations between doctors’ satisfaction with other variables. **Supplementary Figure 1.** The economic regions, hospital levels, and doctors' professional ranks in mainland China.

## Data Availability

All data generated or analyzed during this study are presented in the article and appendix materials.

## Abbreviations

CI: Confidence interval
OR: Odds ratio
PD-L1: Programmed death-ligand 1
PD-1: Programmed cell death protein 1
TCM: Traditional Chinese medicine
