# Supplementary material for: Current status of and barriers to the treatment of advanced-stage liver cancer in China: a questionnaire-based study from the perspective of doctors
Source: BMC Gastroenterol. 2022 Jul 24;22:351. doi: 10.1186/s12876-022-02425-4 (PMC9310466; doi:10.1186/s12876-022-02425-4)
Supplement: Supplementary file 1 — Additional file 1. Supplementary Table 1. Detailed information of the questionnaire. Supplementary Table 2. Correlations between doctors’ satisfaction with other variables. Supplementary Figure 1. The economic regions, hospital levels, and doctors' professional ranks in mainland China. [file 12876_2022_2425_MOESM1_ESM.docx]

**Supplementary Table 1. Detailed information of the questionnaire.**

| Questions and Answers | Number  (n=1,021) | Percentage  (%) |
| --- | --- | --- |
| 1.What is your gender？ | | |
| Male | 588 | 57.6 |
| Female | 433 | 42.4 |
| 2.What is your age? | | |
| 20-31 | 23 | 2.3 |
| 31-40 | 198 | 19.4 |
| 41-50 | 411 | 40.3 |
| >50 | 389 | 38.1 |
| 3.Where do you work? | | |
| East China | 321 | 31.4 |
| Central China | 351 | 34.4 |
| West China | 255 | 25.0 |
| Northeast China | 94 | 9.2 |
| 4. How long have you been working? | | |
| 1-5 years | 35 | 3.4 |
| 6-15 years | 179 | 17.5 |
| 16-25 years | 322 | 31.5 |
| >25 years | 485 | 47.5 |
| 5.What is your professional rank? | | |
| Resident | 27 | 2.6 |
| Attending physician | 136 | 13.3 |
| Deputy chief physician | 356 | 34.9 |
| Chief physician | 502 | 49.2 |
| 6. Which department are you in? | | |
| Hepatology Department | 367 | 36.0 |
| Infection Department | 397 | 38.9 |
| Gastroenterology Department | 178 | 17.4 |
| Oncology Department | 14 | 1.4 |
| Interventional Radiotherapy Department | 2 | 0.2 |
| Others | 37 | 3.6 |
| 7.What is the level of your hospital? | | |
| Secondary and lower hospital | 265 | 26.0 |
| Tertiary hospital | 185 | 18.1 |
| Tertiary first-class hospital | 571 | 55.9 |
| 8. What is the type of your hospital? | | |
| Specialist hospital | 229 | 22.4 |
| General hospital | 792 | 77.6 |
| 9. Do you think it is necessary to prescribe serum markers such as alpha-fetoprotein and abdominal imaging examinations for patients with liver disease during regular outpatient follow-ups? | | |
| Yes | 1017 | 99.6 |
| No | 4 | 0.4 |
| 10. Is your hospital able to conduct further tests to determine the nature of the liver masses? | | |
| Yes | 981 | 96.1 |
| No | 40 | 3.9 |
| 11. What is your recommended hospital when the diagnosis is vague? | | |
| Local hospitals | 153 | 15.0 |
| Provincial capital hospitals | 462 | 45.3 |
| National top hospitals | 406 | 39.8 |
| 12. Is the method of diagnosis enough? | | |
| Yes | 513 | 50.2 |
| No | 508 | 49.8 |
| 13. What is the percentage of first diagnosis? | | |
| 0-20% | 540 | 52.9 |
| 20-40% | 291 | 28.5 |
| 40-60% | 112 | 11.0 |
| >60% | 78 | 7.6 |
| 14. What is the percentage of advanced stage cancer in your practice？ | | |
| 0-20% | 95 | 9.3 |
| 20-40% | 205 | 20.1 |
| 40-60% | 386 | 37.8 |
| >60% | 335 | 32.8 |
| 15. Is the pathological diagnosis important? | | |
| Yes | 519 | 50.8 |
| No | 502 | 49.2 |
| 16. If patients with hepatitis B-related liver cancer are positive for the virus, do they need immediate antiviral treatment? | | |
| Yes | 1004 | 98.3 |
| No | 17 | 1.7 |
| 17. If patients with hepatitis C-related liver cancer are positive for the virus, do they need immediate antiviral treatment? | | |
| Yes | 982 | 96.2 |
| No | 39 | 3.8 |
| 18. Will you actively help a patient with NASH through lifestyle changes, exercise, weight loss and other measures to reduce the possibility of subsequent liver cirrhosis and liver cancer? | | |
| Yes | 1010 | 98.9 |
| No | 11 | 1.1 |
| 19. Who will make the final decisions on the treatment? |  |  |
| Doctors | 141 | 13.8 |
| Patients | 242 | 23.7 |
| Patients’ family | 638 | 62.5 |
| 20. Do you support the disclosure of the patients’ true conditions? |  |  |
| Yes | 632 | 61.9 |
| No | 389 | 38.1 |
| 21. What’s your preferred treatment regimen? |  |  |
| Targeted therapy | 114 | 11.2 |
| Immunotherapy | 12 | 1.2 |
| Target therapy & Immunotherapy | 871 | 85.3 |
| Chemotherapy | 24 | 2.4 |
| 22. What’s your preferred targeted drug? |  |  |
| Sorafenib | 572 | 56.0 |
| Lenvatinib | 344 | 33.7 |
| Rigorfenib | 26 | 2.6 |
| Apatinib | 70 | 6.9 |
| Cabotinib | 9 | 0.9 |
| 23. How do you deal with adverse effects of targeted therapy? |  |  |
| Keeping the dosage and frequency | 356 | 34.9 |
| Reducing the dosage | 533 | 52.2 |
| Interrupted medication on weekends | 48 | 4.7 |
| Take medicine every other day | 84 | 8.2 |
| 24. What’s your preferred immunotherapy drug? |  |  |
| PD-1 | 773 | 75.7 |
| PD-L1 | 210 | 20.6 |
| CTLA-4 | 38 | 3.72 |
| 25. What’s your major drug source? |  |  |
| Imported | 338 | 33.1 |
| Domestic | 154 | 15.1 |
| Available | 529 | 51.8 |
| 26. What’s your major considerations for prescribing? |  |  |
| Insurance | 303 | 29.7 |
| Effectiveness | 494 | 48.4 |
| Cost | 57 | 5.6 |
| Availability | 167 | 16.4 |
| 27. Do you advocate traditional Chinese medicine? |  |  |
| Yes | 634 | 62.1 |
| No | 387 | 37.9 |
| 28. Are drugs sufficient? |  |  |
| Yes | 250 | 24.5 |
| No | 771 | 75.5 |
| 29. Is the price of medication acceptable? | | |
| Yes | 287 | 28.1 |
| No | 734 | 71.9 |
| 30. Is the treatment satisfactory? | | |
| Yes | 239 | 23.4 |
| No | 782 | 76.6 |
| 31. Which of the following treatment models for liver cancer do you expect? | | |
| Complete cure | 570 | 55.8 |
| Long-term treatment | 913 | 89.4 |
| Minimize the suffering of patients and improved the quality of lives | 908 | 88.9 |
| Not to implement painful and risky treatment | 119 | 11.7 |
| 32. What do patients with liver cancer need as far as you are concerned? | | |
| Psychological counseling | 953 | 93.3 |
| Improvement in the quality of life | 958 | 93.8 |
| Relief of economic burden | 952 | 93.2 |
| Promoted early diagnosis rate | 971 | 95.1 |
| More new medications available | 916 | 89.7 |
| 33. How would you like to get access to the updated knowledge on the progress of liver cancer diagnosis and treatment? | | |
| Participating in academic conferences | 1000 | 97.9 |
| Accessing journal literature | 922 | 90.3 |
| Attending video lectures on professional websites | 968 | 94.8 |
| Learning the official policy | 548 | 53.7 |

**Supplementary Table 2. Correlations between doctors’ satisfaction with other variables.**

| **Variables** | **Satisfied** | **Dissatisfied** | **P** | **OR (95% CI)** | **P** |
| --- | --- | --- | --- | --- | --- |
| Economic regions |  |  | 0.015 * |  | 0.016* |
| East China | 59 (24.7%) | 262 (33.5%) |  | Reference |  |
| Central China | 82 (34.3%) | 269 (34.4%) |  | 1.354 (0.930-1.970) | 0.114 |
| West China | 67 (28.0%) | 188 (24.0%) |  | 1.583 (1.064-2.354) | 0.023 |
| Northeast China | 31 (13.0%) | 63 (8.1%) |  | 2.185 (1.306-3.655) | 0.003 |
| Professional ranks |  |  | 0.696 |  | 0.696 |
| Chief physician | 112（46.9%） | 390（49.9%） |  | Reference |  |
| Deputy chief physician | 86（36.0%） | 270（34.5%） |  | 1.109 (0.805-1.529) | 0.527 |
| Attending physician and resident | 41（17.2%） | 122（15.6%） |  | 1.170 (0.775-1.766) | 0.454 |
| Level of hospital |  |  | 0.240 |  | 0.241 |
| Tertiary first-class hospital | 127（53.1%） | 444（56.8%） |  | Reference |  |
| Tertiary hospital | 40（16.7%） | 145（18.5%） |  | 0.964 (0.645-1.441) | 0.860 |
| Secondary and lower hospital | 72（30.1%） | 193（24.7%） |  | 1.304 (0.933-1.823) | 0.120 |
| Are drugs sufficient? |  |  | <0.001* |  |  |
| Yes | 111（46.4%） | 139（17.8%） |  | Reference |  |
| No | 128（53.6%） | 643（82.2%） |  | 0.249 (0.182-0.341) | <0.001* |
| What’s your major drug source? |  |  | 0.055 |  | 0.056 |
| Imported | 85（35.6%） | 253（32.4%） |  | Reference |  |
| Domestic | 109（45.6%） | 420（53.7%） |  | 0.772 (0.559-1.068) | 0.118 |
| Available | 45（18.8%） | 109（13.9%） |  | 1.229 (0.803-1.880) | 0.342 |
| What is the percentage of advanced stage in your practice? |  |  | <0.001* |  | <0.001* |
| <40% | 104（43.5%） | 196（25.1%） |  | Reference |  |
| 40-60% | 86（36.0%） | 300（38.4%） |  | 0.540 (0.385-0.757) | <0.001 |
| >60% | 49（20.5%） | 286（36.6%） |  | 0.323 (0.220-0.475) | <0.001 |
| What is the percentage of first diagnosis? |  |  | 0.306 |  | 0.307 |
| <20% | 136（56.9%） | 404（51.7%） |  | Reference |  |
| 20-40% | 65（27.2%） | 226（28.9%） |  | 0.854 (0.610-1.197) | 0.361 |
| >40% | 38（15.9%） | 152（19.4%） |  | 0743 (0.495-1.114) | 0.150 |
| Is the method of diagnosis enough? |  |  | <0.001* |  |  |
| Yes | 146（61.1%） | 367（46.9%） |  | Reference |  |
| No | 93（38.9%） | 415（53.1%） |  | 0.563 (0.419-0.757) | <0.001* |
| Do you advocate traditional Chinese medicine? |  |  | 0.484 |  |  |
| Yes | 153（64.0%） | 481（61.5%） |  | Reference |  |
| No | 86（36.0%） | 301（38.5%） |  | 0.898 (0.665-1.214) | 0.484 |
| What’s your preferred immunotherapy drug? |  |  | 0.144 |  | 0.147 |
| PD-1 | 192（80.3%） | 581（74.3%） |  | Reference |  |
| PD-L1 | 41（17.2%） | 169（21.6%） |  | 0.734 (0.503-1.072) | 0.109 |
| CTLA-4 | 6（2.5%） | 32（4.1%） |  | 0.567 (0.234-1.378) | 0.211 |
| What is your preferred targeted drug? |  |  | 0.065 |  |  |
| First-line drugs | 222（92.9%） | 694（88.7%） |  | Reference |  |
| Second-line drugs | 17（7.1%） | 88（11.3%） |  | 0.604 (0.352-1.037) | 0.068 |
| How do you deal with adverse effects of targeted therapy? |  |  | 0.003* |  | 0.004* |
| Keeping the dosage and frequency | 105（43.9%） | 251（32.1%） |  | Reference |  |
| Reducing the dosage | 108（45.2%） | 425（54.3%） |  | 0.607 (0.445-0.829) | 0.003 |
| Intermittent medication | 26（10.9%） | 106（13.6%） |  | 0.586 (0.361-0.953) | 0.031 |
| What are your major considerations for prescribing? |  |  | 0.226 |  | 0.228 |
| Cost or insurance | 84（35.1%） | 276（35.3%） |  | Reference |  |
| Effectiveness | 124（51.9%） | 370（47.3%） |  | 1.101 (0.801-1.513) | 0.552 |
| Availability | 31（13.0%） | 136（17.4%） |  | 0.749 (0.473-1.187) | 0.218 |
| What’s your preferred treatment regimen? |  |  | 0.603 |  | 0.641 |
| Targeted therapy | 25（10.5%） | 89（11.4%） |  | Reference |  |
| Immunotherapy | 1（0.4%） | 11（1.4%） |  | 0.324 (0.040-2.629) | 0.291 |
| Target therapy & Immunotherapy | 208（87.0%） | 663（84.8%） |  | 1.117 (0.698-1.787) | 0.645 |
| Chemotherapy | 5（2.1%） | 19（2.4%） |  | 0.937 (0.318-2.760) | 0.906 |
| Do you support the disclosure of the patients’ true conditions? |  |  | 0.032* |  |  |
| No | 77（32.2%） | 312（39.9%） |  | Reference |  |
| Yes | 162（67.8%） | 470（60.1%） |  | 1.397 (1.028-1.898) | 0.033* |
| Who will make the final decisions on treatment? |  |  | 0.796 |  | 0.797 |
| Doctors | 36（15.1%） | 105（13.4%） |  | Reference |  |
| Patients | 57（23.8%） | 185（23.7%） |  | 0.899 (0.555-1.454) | 0.663 |
| Patients’ family | 146（61.1%） | 492（62.9%） |  | 0.866 (0.568-1.319) | 0.502 |
| Is the pathological diagnosis important? |  |  | 0.160 |  |  |
| Yes | 131（54.8%） | 388（49.6%） |  | Reference |  |
| No | 108（45.2%） | 394（50.4%） |  | 0.812 (0.607-1.086) | 0.160 |
| What is your recommended hospital when the diagnosis is vague? |  |  | 0.135 |  | 0.136 |
| Local hospitals | 35（14.6%） | 118（15.1%） |  | Reference |  |
| Provincial capital hospitals | 121（50.6%） | 341（43.6%） |  | 1.196 (0.778-1.840) | 0.414 |
| National top hospitals | 83（34.7%） | 323（41.3%） |  | 0.866 (0.554-1.356) | 0.530 |

Notes: P values in the fourth column belong to χ2 analysis; P values in the sixth column belong to univariate logistic analysis. * indicates statistical significance (P<0.05).

**Supplementary Figure**

**
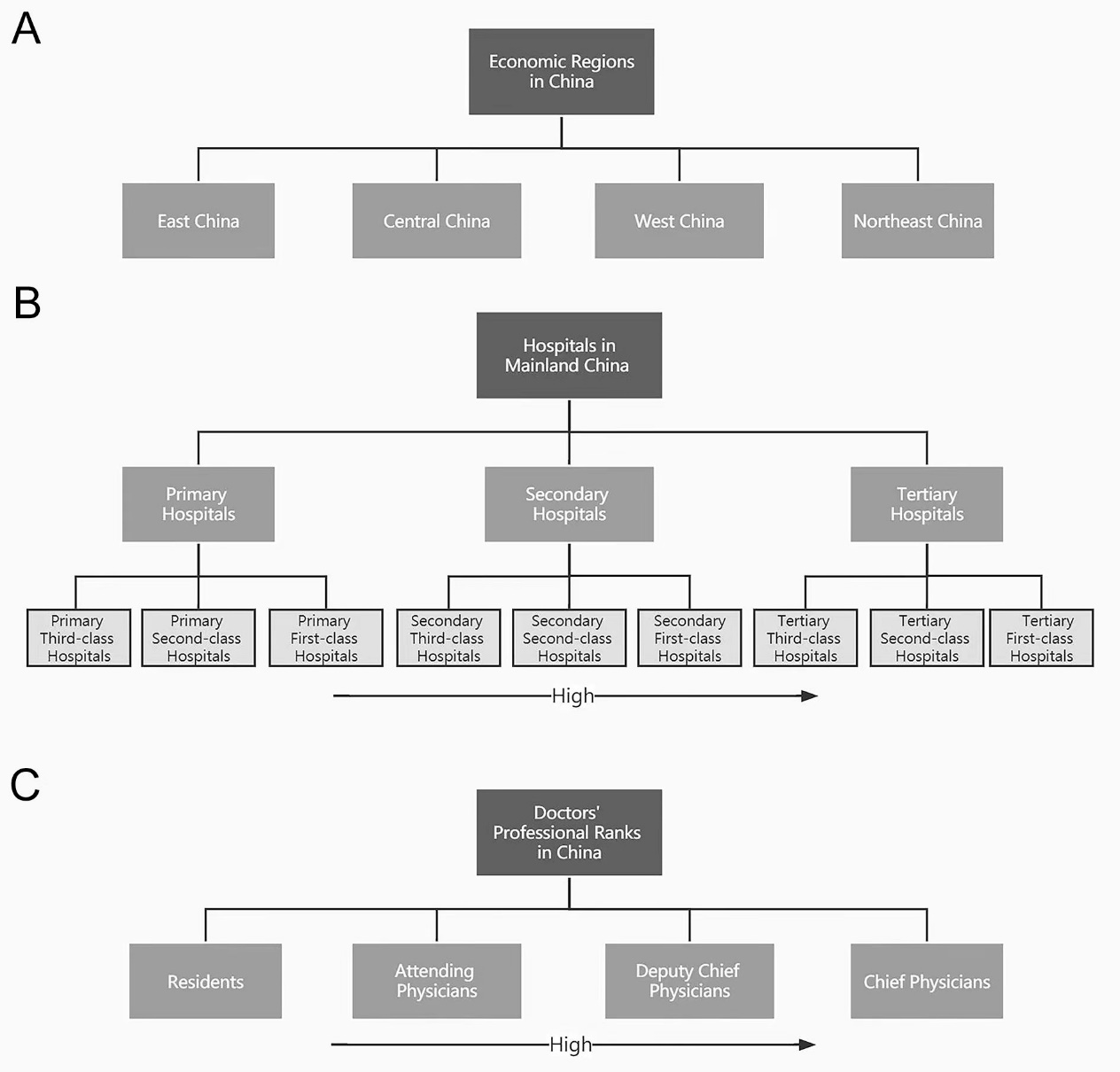
**

Supplementary Figure 1. Illustration of the economic regions (A), hospital levels (B), and doctors' professional ranks (C) in mainland China. The arrows indicated higher hospital level in panel B and higher professional rank in panel C.
